# Supplementary material for: Identification of NPC1 as the target of U18666A, an inhibitor of lysosomal cholesterol export and Ebola infection
Source: eLife. 2015 Dec 8;4:e12177. doi: 10.7554/eLife.12177 (PMC4718804; doi:10.7554/eLife.12177)
Supplement: Supplementary file 1. — DOI: http://dx.doi.org/10.7554/eLife.12177.010 [file elife-12177-supp1.doc]

**Supplemental File 1**

Six schemes showing the synthesis of derivatives of U18666A**.**

**Scheme 1. Synthesis of compound A**

Reagents and conditions: a) HCO_2_H, 110^o^C; b) NaH, THF, reflux.

**Compound 2:** To a solution of dehydroepiandrosterone **1** (288 mg, 1.0 mmol) in 1.0 ml of HCO_2_H was added dimethylamine (3.0 mmol) at room temperature. The solution was heated to 110^o^C and stirred overnight. After cooling to room temperature, the mixture was poured into aq. NaOH (6N, 10 ml) at 0^o^C and extracted with EtOAc (3 × 20 ml). The combined organic layers were washed with brine (3 × 3 ml), dried (Na_2_SO_4_), and concentrated. The residue was purified by column chromatography (silica gel; MeOH:CH_2_Cl_2_ = 1:10) to yield compound **2** (120 mg, 0.37 mmol, 38%).

**^1^H NMR** (400 MHz, CDCl_3_) δ 5.32 (d, *J* = 4.1 Hz, 1H), 3.53-3.45 (m, 1H), 2.23 (s, 6H), 2.29-2.17 (m, 3H), 2.05-1.75 (m, 6H), 1.63-1.40 (m, 6H), 1.27-1.10 (m, 2H), 1.10-0.97 (m, 2H), 0.98 (s, 3H), 0.88 (ddd, *J* = 12.0, 12.0, 4.0 Hz 1H), 0.82 (s, 3H); **^13^C NMR** (100 MHz, CDCl_3_) δ 140.76, 121.39, 77.88, 71.65, 54.21, 49.75, 45.92, 42.83, 42.23, 39.51, 37.19, 36.40, 31.79, 31.59, 28.98, 23.49, 21.01, 19.34, 11.80; **ESI MS:** [M+H]^+^ calcd. for C_21_H_36_NO, 318.2, found 318.2.

**Compound A:** To solution of compound **2** (10 mg, 0.03 mmol) in THF (2 ml) was added NaH (7 mg, 0.3 mmol) at room temperature. The solution was stirred for 1 hr at room temperature, followed by the addition of 3-ethyl-1-iodopentane (22 mg, 0.09 mmol). The solution was refluxed overnight, cooled to room temperature, quenched with saturated aq. NH_4_Cl (5 ml), and extracted with EtOAc (3 × 10 ml). The combined organic layers were washed with brine (3 × 3 ml), dried (Na_2_SO_4_), and concentrated. The residue was purified by column chromatography (silica gel; MeOH:CH_2_Cl_2_ = 1:10) to afford compound **A** (4.5 mg, 0.01 mmol, 37%).

**^1^H NMR** (400 MHz, CDCl_3_) δ 5.33 (d, *J* = 4.2 Hz, 1H), 3.45 (t, *J* = 7.2 Hz, 2H), 3.20-3.06 (m, 1H), 2.34 (s, 6H), 2.40-2.25 (m, 2H), 2.18 (app. t, *J* = 12.4 Hz, 1H), 2.09-1.80 (m, 6H), 1.70-1.38 (m, 8H), 1.31-1.17 (m, 7H), 1.09-0.98 (m, 2H), 0.99 (s, 3H), 0.89 (s, 3H), 0.95-0.80 (m, 1H), 0.84 (t, *J* = 6.8 Hz, 6H); **^13^C NMR** (100 MHz, CDCl_3_) δ 141.16, 121.02, 78.88, 77.94, 66.49, 54.20, 49.79, 45.74, 42.91, 39.45, 39.15, 37.40, 37.25, 36.82, 33.24, 31.77, 31.59, 28.62, 28.44, 25.46, 23.43, 20.97, 19.34, 12.15, 10.74; **ESI MS:** [M+H]^+^ calcd. for C_28_H_50_NO, 416.3, found 416.4; **[α]_D_: +**4.96 (c = 0.22, CHCl_3_); calculated ClogP and pKa: 4.69 and 10.21.

**Scheme 2. Synthesis of compound B**

Reagents and conditions: a) ethylene glycol, *p*-TsOH, benzene, reflux; b) NaH, THF, (2-bromoethoxy)(*tert*-butyl)dimethylsilane, reflux; c) aq. HCl (conc.), MeOH, rt; d) MsCl, pyridine, CH_2_Cl_2_, rt; e) N-propylprop-2-yn-1-amine, toluene, reflux; f) 3-azidopyridine, CuSO_4_·5H_2_O, Na-ascorbate, THF / H_2_O, rt. Abbreviations: Ms, methanesulfonyl; Ts, toluenesulfonyl.

**Compound 3**: To a solution of dehydroepiandrosterone (25 g, 86.8 mmol) and *p*-TsOH·H_2_O (0.824 g, 4.3 mmol) in benzene (200 ml) was added ethylene glycol (16.1 g, 260.4 mmol) dropwise. The mixture was refluxed under a Dean-Stark trap for 48 hr. The reaction mixture was cooled to room temperature, diluted with EtOAc (200 ml), and washed with saturated aq. NaHCO_3_ (3 × 15 ml) and brine (3 × 10 ml). The organic phase was dried (Na_2_SO_4_) and concentrated. The residue was recrystallized from EtOAc:Hexanes (1:3) to afford compound **3** (26 g, 78.3 mmol, 90%). [The synthesis of compound **3**

(17,17-ethylenodioxy-5-androsten-3β-ol) has been described: Calogeropoulou, T.; Avlonitis, N.; Minas, V.; Alexi, X.; Pantzou, A.; Charalampopoulos, I.; Zervou, M.; Vergou, V.; Katsanou, E.; Lazaridis, I.; Alexis, M. N.; Gravanis, A. *J. Med. Chem.* **2009**, *52*, 6569-6587]

**^1^H NMR** (400 MHz, CDCl_3_) δ 5.34-5.33 (m, 1H), 3.98-3.80 (m, 4H), 3.58-3.46 (m, 1H), 2.34-2.16 (m, 2H), 2.05-1.95 (m, 2H), 1.90-1.73 (m, 3H), 1.72-1.62 (m, 1H), 1.60-1.34 (m, 8H), 1.25 (dddd, *J* = 12.0, 12.0, 12.0, 8.0 Hz, 1H), 1.15 (ddd, *J* = 12.0, 12.0, 4.0 Hz, 1H), 1.02-0.92 (m, 1H), 1.00 (s, 3H), 0.85 (s, 3H); **^13^C NMR** (125 MHz, CDCl_3_) δ 140.99, 121.64, 119.75, 71.81, 65.41, 64.79, 50.83, 50.17, 45.96, 42.48, 37.50, 36.78, 34.44, 32.40, 31.81, 31.48, 30.81, 23.02, 20.73, 19.68, 14.47; **ESI MS:** [M+H]^+^ calcd. for C_21_H_33_O_3_, 333.2, found 333.2.

**Compound 4**: To a solution of compound **3** (66 mg, 0.2 mmol) in THF (4 ml) was added NaH (50 mg, 2.0 mmol), and the mixture was refluxed for 1 hr. After cooling to room temperature, (2-bromoethoxy)(tert-butyl)dimethylsilane (189 mg, 0.8 mmol) was added to this solution. The mixture was refluxed overnight while stirring, cooled to room temperature, quenched with saturated aq. NH_4_Cl (5 ml), and extracted with EtOAc (3 × 10 ml). The combined organic layers were washed with brine (2 × 3 ml), dried (Na_2_SO_4_), and concentrated. The residue was purified by column chromatography (silica gel; EtOAc:Hexanes = 1:10) to afford compound **4** (44 mg, 0.089 mmol, 45%).

**^1^H NMR** (500 MHz, CDCl_3_) δ 5.39-5.32 (m, 1H), 4.00-3.82 (m, 4H), 3.75 (t, *J* = 6.0 Hz, 2H), 3.55 (t, *J* = 6.0 Hz, 2H), 3.27-3.12 (m, 1H), 2.38 (br.d, *J* = 12.0 Hz, 1H), 2.19 (app. t, *J* = 12.3 Hz, 1H), 2.05-1.98 (m, 2H), 1.95-1.75 (m, 3H), 1.73-1.65 (m, 1H), 1.65-1.52 (m, 3H), 1.52-1.36 (m, 4H), 1.33-1.20 (m, 2H), 1.01 (s, 3H), 1.07-0.94 (m, 2H), 0.90 (s, 9H), 0.87 (s, 3H), 0.08 (s, 6H); **^13^C NMR** (125 MHz, CDCl_3_) δ 141.18, 121.47, 119.74, 79.53, 69.54, 65.42, 64.79, 63.20, 50.85, 50.25, 45.96, 39.37, 37.48, 37.13, 34.44, 32.40, 31.51, 30.82, 28.64, 26.18, 23.01, 20.71, 19.65, 18.63, 14.45; **ESI MS:** [M+H]^+^ calcd. for C_29_H_51_O_4_Si, 491.3, found 491.4.

**Compound 5**: To solution of compound **4** (44 mg, 0.08 mmol) in MeOH (4 ml) was added aq. HCl (conc., 0.2 ml). After stirring for 1 hr at room temperature, the mixture was diluted with EtOAc (20 ml) and washed with saturated aq. NaHCO_3_ (2 × 5 ml) and brine (2 × 3 ml). The organic layer was dried (Na_2_SO_4_) and concentrated. The residue was purified by column chromatography (silica gel; EtOAc:Hexanes = 1:3) to afford compound **5** (25 mg, 0.075 mmol, 83%).

**^1^H NMR** (400 MHz, CDCl_3_) δ 5.33 (d, *J* = 4.7 Hz, 1H), 3.77-3.62 (m, 2H), 3.60-3.48 (m, 2H), 3.23-3.13 (m, 1H), 2.46-2.29 (m, 2H), 2.17 (app. t, *J* = 12.2 Hz, 1H), 2.12-1.97 (m, 2H), 1.95-1.75 (m, 4H), 1.69-1.54 (m, 3H), 1.54-1.36 (m, 3H), 1.30-1.17 (m, 2H), 1.07-0.91 (m, 2H), 0.98 (s, 3H), 0.84 (s, 3H); **^13^C NMR** (100 MHz, CDCl_3_) δ 221.09, 140.90, 120.91, 79.17, 69.01, 61.99, 51.70, 50.21, 47.49, 39.02, 37.05, 36.94 35.79, 31.43, 31.38, 30.76, 28.27, 21.83, 20.31, 19.36, 13.51; **ESI MS:** [M+H]^+^ calcd. for C_21_H_33_O_3_, 333.2, found 333.3.

**Compound 6**: To a solution of compound **5** (6.85 g, 20.6 mmol) and pyridine (4.88 g, 61.8 mmol) in CH_2_Cl_2_ (100 ml) was added MsCl (3.05 g, 26.8 mmol) at 0^o^C. The mixture was stirred for 48 hr at room temperature; then diluted with CH_2_Cl_2_ (150 ml), and washed with aq. HCl (2N, 3 × 20 ml) and saturated aq. NaHCO_3_ (3 × 15 ml). The organic layer was dried (Na_2_SO_4_) and concentrated. Recrystallization of the residue from EtOAc:Hexanes (1:3) afforded compound **6** (7.1 g, 17.3 mmol, 84%).

**^1^H NMR** (500 MHz, CDCl_3_) δ 5.39 (d, *J* = 4.9 Hz, 1H), 4.48-4.23 (m, 2H), 3.78-3.73 (m, 2H), 3.27-3.22 (m, 1H), 3.07 (s, 3H), 2.47 (dd, *J* = 17.5, 7.5 Hz, 1H), 2.43-2.35 (m, 1H), 2.22 (app. t, *J* = 12.5 Hz, 1H), 2.16-2.05 (m, 2H), 1.97-1.83 (m, 4H), 1.75-1.60 (m, 3H), 1.60-1.43 (m, 3H), 1.35-1.25 (m, 2H), 1.12-0.96 (m, 2H), 1.03 (s, 3H), 0.89 (s, 3H); **^13^C NMR** (125 MHz, CDCl_3_) δ 194.67, 140.87, 121.46, 79.74, 69.91, 66.02, 51.96, 50.45, 47.78, 39.14, 37.97, 37.24, 36.09, 31.70, 31.64, 31.03, 28.43, 22.12, 20.59, 19.64, 13.79; **ESI MS:** [M+H]^+^ calcd. for C_22_H_35_O_5_S, 411.2, found 411.2.

**Compound 7**: To a solution of compound **6** (1.156 g, 2.82 mmol) in toluene (20 ml) was added N-propylprop-2-yn-1-amine (1.09 g, 11.3 mmol). The mixture was stirred for 48 hr at 120^o^C, cooled to room temperature, diluted with EtOAc (100 ml), and washed with H_2_O (3 × 10 ml). The organic phase was dried (Na_2_SO_4_) and concentrated. The residue was purified by flash chromatography (silica gel; Hexanes:EtOAc = 3:1) to yield compound **7** (0.934 g, 2.27 mmol, 81%).

**^1^H NMR** (500 MHz, CDCl_3_) δ 5.37 (s, 1H), 3.58 (t, *J* = 6.2 Hz, 2H), 3.46 (s, 2H), 3.25-3.12 (m, 1H), 2.71 (t, *J* = 6.1 Hz, 2H), 2.43-2.50 (m, 3H), 2.43-2.35 (m, 1H), 2.22 (t, *J* = 12.5 Hz, 1H), 2.18 (t, *J* = 2.5 Hz, 1 H), 2.16-2.03 (m, 2H), 2.01-1.80 (m, 4H), 1.75-1.42 (m, 8H), 1.1.33-1.23 (m, 2H), 1.08-0.88 (m, 2H), 1.00 (s, 3H), (m, 5H), 0.92 (t, *J* = 6.7 Hz), 0.89 (s, 3H); **^13^C NMR** (125 MHz, CDCl_3_) δ 221.50, 141.50, 120.96, 104.99, 79.39, 72.96, 66.65, 56.38, 53.53, 52.00, 50.51, 47.80, 42.93, 39.30, 37.41, 37.26, 36.10, 31.72, 31.67, 31.06, 28.52, 22.12, 20.94, 20.59, 19.66, 13.79, 12.10; **ESI MS:** [M+H]^+^ calcd. for C_27_H_42_NO_2_, 412.3, found 412.1.

**Compound B:** To a solution of 3-azidopyridine (1.62 g, 13.5 mmol) and compound **7** (3.7 g, 9.0 mmol) in THF (50 ml) was added a solution of CuSO_4_·5H_2_O (450 mg, 1.8 mmol) in H_2_O (5 ml) and a solution of Na-ascorbate (178 mg, 0.9 mmol) in H_2_O (5 ml). The mixture was stirred for 2 hr at room temperature, followed by removal of THF in vacuo. After addition of H_2_O (20 ml), the solution was extracted with CH_2_Cl_2_ (3 × 36 ml). The combined organic layers were dried (Na_2_SO_4_) and concentrated. The residue was purified by flash chromatography (silica gel; EtOAc:MeOH = 5:1) to yield compound **B** (4.48 g, 8.43 mmol, 94%).

**^1^H NMR** (500 MHz, CDCl_3_) δ8.98 (s, 1H), 8.65 (d, *J* = 4.5 Hz, 1H), 8.13 (dd, *J* = 7.7, 1.5 Hz, 1H), 8.05 (s, 1H), 7.47 (dd, *J* = 7.7, 4.5 Hz, 1H), 5.32 (d, *J* = 3.0 Hz, 1H), 3.93 (s, 2H), 3.59 (app. t, *J* = 7.5 Hz, 2H), 3.21-3.07 (m, 1H), 2.70 (app. t, *J* = 7.5 Hz, 2H), 2.48 (app. t, *J* = 7.5 Hz, 2H ), 2.41 (dd, *J* = 20.0, 10.0 Hz, 1H ), 2.35 (br. d, *J* = 11.5 Hz, 1H), 2.16 (t, *J* = 11.5 Hz, 1H), 2.12-1.97 (m, 2H), 1.95-1.76 (m, 4H), 1.73-1.57 (m, 3H), 1.57-1.47 (m, 3H), 1.47-1.35 (m, 2H), 1.32-1.16 (m, 2H), 0.97 (s, 3H), 1.07-0.90 (m, 2H), 0.86 (t, *J* = 7.0 Hz, 3H), 0.84 (s, 3H); **^13^C NMR** (125 MHz, CDCl_3_) δ 221.30, 149.91, 147.41, 141.60, 141.26, 134.02, 128.07, 124.44, 121.07, 120.88, 79.39, 66.80, 60.58, 56.77, 53.72, 51.95, 50.46, 49.62, 47.73, 39.36, 37.33, 37.21, 36.04, 31.68, 31.64, 31.00, 28.60, 22.08, 21.27, 20.61, 20.55, 19.62, 14.42, 13.76, 12.06; **ESI MS:** [M+H]^+^ calcd. for C_32_H_46_N_5_O_2_, 532.3, found 532.0; **[α]_D_: +**5.45 (c = 0.44, CHCl_3_); calculated ClogP and pKa: 2.54 and 7.17.

**Scheme 3. Synthesis of compound C**

Reagents and conditions: a) EDCI, DMAP, CH_2_Cl_2_, rt; b) compound **7**, CuSO_4_·5H_2_O, Na-ascorbate, THF/H_2_O, rt. Abbreviations: DMAP, 4-(dimethylamino)pyridine; EDCI,

1-ethyl-3-(3-dimethylaminopropyl)carbodiimide.

**Compound 10:** To a solution of *D*-biotin (**8**, 0.7 g, 2.8 mmol) and 6-azidohexan-1-ol (**9**, 400 mg, 2.8 mmol) in CH_2_Cl_2_ (10 ml) was added EDCI (2 g, 10.5 mmol) and DMAP (36 mg, 0.3 mmol) at 0^o^C. The solution was warmed to room temperature and stirred for 48 hr. The solution was diluted with EtOAc (50 ml) and washed with brine (3 × 8 ml). The organic layer was dried (Na_2_SO_4_) and concentrated. The residue was purified by flash chromatography (silica gel; EtOAc:Hexanes = 1:1) to provide compound **10** (0.84 g, 2.27 mmol, 79%).

**^1^H NMR** (400 MHz, CDCl_3_) δ 6.05 (s, 1H), 5.69 (s, 1H), 4.46 (dd, *J* = 7.2, 5.2 Hz, 1H), 4.27 (dd, *J* = 7.2, 5.2 Hz, 1H), 4.02 (t, *J* = 6.4 Hz, 2H), 3.24 (t, *J* = 6.4 Hz, 2H), 3.12 (dd, *J* = 12.8, 7.6 Hz, 1H), 2.87 (dd, *J* = 12.8, 4.8 Hz, 1H), 2.70 (d, *J* = 12.8 Hz, 1H), 2.29 (t, *J* = 7.6 Hz, 2H), 1.77-1.49 (m, 8H), 1.47-1.27 (m, 6H); **^13^C NMR** (100 MHz, CDCl_3_) δ 173.71, 163.58, 64.23, 61.91, 60.10, 55.42, 51.30, 40.53, 33.90, 28.70, 28.46, 28.36, 28.24, 26.34, 25.51, 24.78; **ESI MS:** [M+H]^+^ calcd. for C_16_H_28_N_5_O_3_S, 370.1, found 370.0. [The spectral data is in full agreement with those published for compound **10**: Prasad, G; Amoroso, J. W.; Borketey, L. S.; Schnarr, N. A.; *Org. Biomol. Chem.* **2012**, *10*, 1992-2002]

**Compound C:** To a solution of compounds **10** (1.068 g, 2.89 mmol) and **7** (1.19 g, 2.89 mmol) in THF (30 ml) was added a solution of CuSO_4_·5H_2_O (72 mg, 0.29 mmol) in H_2_O (3 ml) and a solution of Na-ascorbate (30 mg, 0.15 mmol) in H_2_O (3 ml). The mixture was stirred overnight at room temperature, followed by removal of THF in vacuo. After the addition of H_2_O (15 ml), the mixture was extracted with CH_2_Cl_2_ (3 × 20 ml) and the combined organic layers were dried (Na_2_SO_4_) and concentrated. The residue was purified by flash chromatography (silica gel; EtOAc:MeOH = 5:1) to give compound **C** (2.1 g, 2.69 mmol, 93%).

**^1^H NMR** (500 MHz, CDCl_3_) δ 7.47 (s, 1H), 6.06 (s, 1H), 5.74 (s, 1H), 5.32 (d, *J* = 4.5 Hz, 1H), 4.46 (dd, *J* = 7.0, 5.5 Hz, 1H), 4.36-4.22 (m, 3H), 4.00 (t, *J* = 6.5 Hz, 2H), 3.81 (s, 2H), 3.55 (t, *J* = 6.0 Hz, 2H), 3.18-3.05 (m, 2H), 2.85 (dd, *J* = 12.5, 5.0 Hz, 1H), 2.69 (d, *J* = 12.5 Hz, 1H), 2.64 (t, *J* = 6.0 Hz, 2H), 2.47-2.37 (m, 3H), 2.33 (dd, *J* = 13.5, 2.5 Hz, 1H), 2.27 (t, *J* = 7.5 Hz, 2H), 2.14 (t, *J* = 11.5 Hz, 1H), 2.06 (t, *J* = 9.0 Hz, 2H), 1.95-1.76 (m, 4H), 1.74-1.28 (m, 20H), 1.28-1.17 (m, 2H), 0.97 (s, 3H), 1.06-0.91 (m, 2H), 0.84 (s, 3H), 0.82 (t, *J* = 7.0 Hz, 3H); **^13^C NMR** (125 MHz, CDCl_3_) δ221.37, 173.91, 164.00, 141.32, 122.66, 122.65, 121.01, 79.32, 66.61, 64.33, 62.17, 60.34, 56.53, 55.74, 53.74, 53.56, 51.94, 50.45, 50.28, 49.67, 47.74, 40.76, 39.33, 37.34, 37.20, 36.05, 34.14, 31.68, 31.01, 30.41, 28.63, 28.55, 28.48, 28.46, 26.36, 25.64, 25.04, 22.08, 20.55, 20.48, 19.62, 13.76, 12.06; **ESI MS:** [M+H]^+^ calcd. for C_43_H_69_N_6_O_5_S 781.5, found 781.6; **[α]_D_: +**24.86 (c = 0.37, CHCl_3_); calculated ClogP and pKa: 3.48 and 7.20.

**Scheme 4. Synthesis of compound D**

Reagents and conditions: a) NaH, THF, reflux; b) *p*-TsOH, acetone, H_2_O, room temperature. Abbreviations: Ts, toluenesulfonyl.

**Compound 12:** To solution of **compound 3** (33 mg, 0.1 mmol) in THF (4 ml) was added NaH (30 mg, 1.0 mmol) and the mixture was stirred under reflux for 1 hr. After cooling to room temperature, 3-ethyl-1-iodopentane (113 mg, 0.5 mmol) was added and the solution was stirred overnight under reflux, cooled to room temperature, and quenched with saturated aq. NH_4_Cl (5 ml). After extraction with EtOAc (3 × 10 ml), the combined organic layers were washed with brine (2 × 3 ml), dried (Na_2_SO_4_), and concentrated. The residue was purified by column chromatography (silica gel; EtOAc:Hexanes = 1:5) to give compound **12** (15 mg, 0.035 mmol, 35%).

**^1^H NMR** (500 MHz, CDCl_3_) δ 5.36-5.34 (m, 1H), 4.00-3.83 (m, 4H), 3.47 (dt, *J* = 7.5, 2.5 Hz, 2H), 3.22-3.06 (m, 1H), 2.37 (dd, *J* = 13.0, 2.0 Hz, 1H), 2.20 (app. t, *J* = 11.5 Hz, 1H), 2.07-1.97 (m, 2H), 1.94-1.76 (m, *3*H), 1.75-1.65 (m, 1H), 1.64-1.37 (m, 10H), 1.36-1.18 (m, 6H), 1.01 (s, 3H), 1.10-0.94 (m, 2H), 0.87 (s, 3H), 0.85 (t, *J* = 7.0 Hz, 6H); **^13^C NMR** (125 MHz, CDCl_3_) δ 141.36, 121.38, 119.76, 79.12, 66.76, 65.43, 64.80, 50.87, 50.27, 45.98, 39.45, 37.63, 37.55, 37.19, 34.45, 33.48, 32.41, 31.52, 30.83, 28.72, 25.70, 23.02, 20.71, 19.66, 14.46, 10.99; **ESI MS:** [M+H]^+^ calcd. for C_28_H_47_O_3_, 431.1, found 431.1.

**Compound D:** To a solution of compound **12** (8 mg, 0.025 mmol) in acetone (2 ml) and water (0.5 ml) was added *p*-TsOH (10 mg). The solution was stirred overnight at room temperature and concentrated. The residue was purified by column chromatography (silica gel; EtOAc:Hexanes = 1:5) to afford compound **D** (7 mg, 0.018 mmol, 92%).

**^1^H NMR** (400 MHz, CDCl_3_) δ 5.39 (d, *J* = 4.8 Hz, 1H), 3.48 (t, *J* = 7.6 Hz, 2H), 3.26-3.05 (m, 1H), 2.46 (dd, *J* = 19.2, 8.8 Hz, 1H), 2.38 (dd, *J* = 12.8, 2.8 Hz, 1H), 2.20 (app. t, *J* = 12.0 Hz, 1H), 2.15-2.02 (m, 2H), 2.05-1.79 (m, 4H), 1.78-1.62 (m, 3H), 1.62-1.40 (m, 6H), 1.39-1.20 (m, 6H), 1.02 (s, 3H), 1.14-0.96 (m, 2H), 0.88 (s, 3H), 0.84 (t, *J* = 7.2 Hz, 6H); **^13^C NMR** (100 MHz, CDCl_3_) δ 221.22, 141.40, 120.59, 78.78, 66.54, 51.77, 50.30, 47.55, 39.21, 37.40, 37.22, 37.04, 35.84, 33.24, 31.49, 31.43, 30.82, 28.41, 25.47, 21.87, 20.34, 19.41, 13.54, 10.75; **ESI MS:** [M+H]^+^ calcd. for C_26_H_43_O_2_, 387.3, found 387.2; **[α]_D_: +**4.0 (c = 0.3, CHCl_3_); calculated ClogP: 3.49.

**Scheme 5. Synthesis of compound E**

Reagents and conditions: a) NaH, THF, reflux.

**Compound E:** To solution of **cyclohexanol** (**13**, 300 mg, 3.0 mmol) in THF (30 ml) was added NaH (151 mg, 6.0 mmol) at room temperature. After stirring for 1 hr at room temperature, 2-chloro-N,N-diethylethanamine (**14**, 172 mg, 1.0 mmol) was added and the solution was stirred overnight under reflux, cooled to room temperature, and quenched with saturated aq. NH_4_Cl (5 ml). After extraction with EtOAc (3 × 10 ml), the combined organic layers were washed with brine (2 × 3 ml), dried (Na_2_SO_4_), and concentrated. The residue was purified by column chromatography (silica gel; CH_2_Cl_2_:MeOH = 10:1) to afford compound **E** (160 mg, 0.80 mmol, 80%).

**^1^H NMR** (400 MHz, CDCl_3_) δ 3.51 (t, *J* = 6.8 Hz, 2H), 3.25-3.15 (m, 1H), 2.62 (t, *J* = 6.8 Hz, 2H), 2.54 (q, *J* = 7.2 Hz, 4H), 1.93-1.84 (m, 2H), 1.76-1.65 (m, 2H), 1.54-1.45 (m, 1H), 1.31-1.09 (m, 5H), 1.00 (t, *J* = 7.2 Hz, 6H); **^13^C NMR** (100 MHz, CDCl_3_) δ 77.79, 66.34, 52.74, 47.70, 32.26, 25.82, 24.17, 11.78; **ESI MS:** [M+H]^+^ calcd. for C_12_H_26_NO 200.1, found 200.1; calculated ClogP and pKa: 3.31 and 9.41.

**Scheme 6. Synthesis of U-X Photocrosslinker**


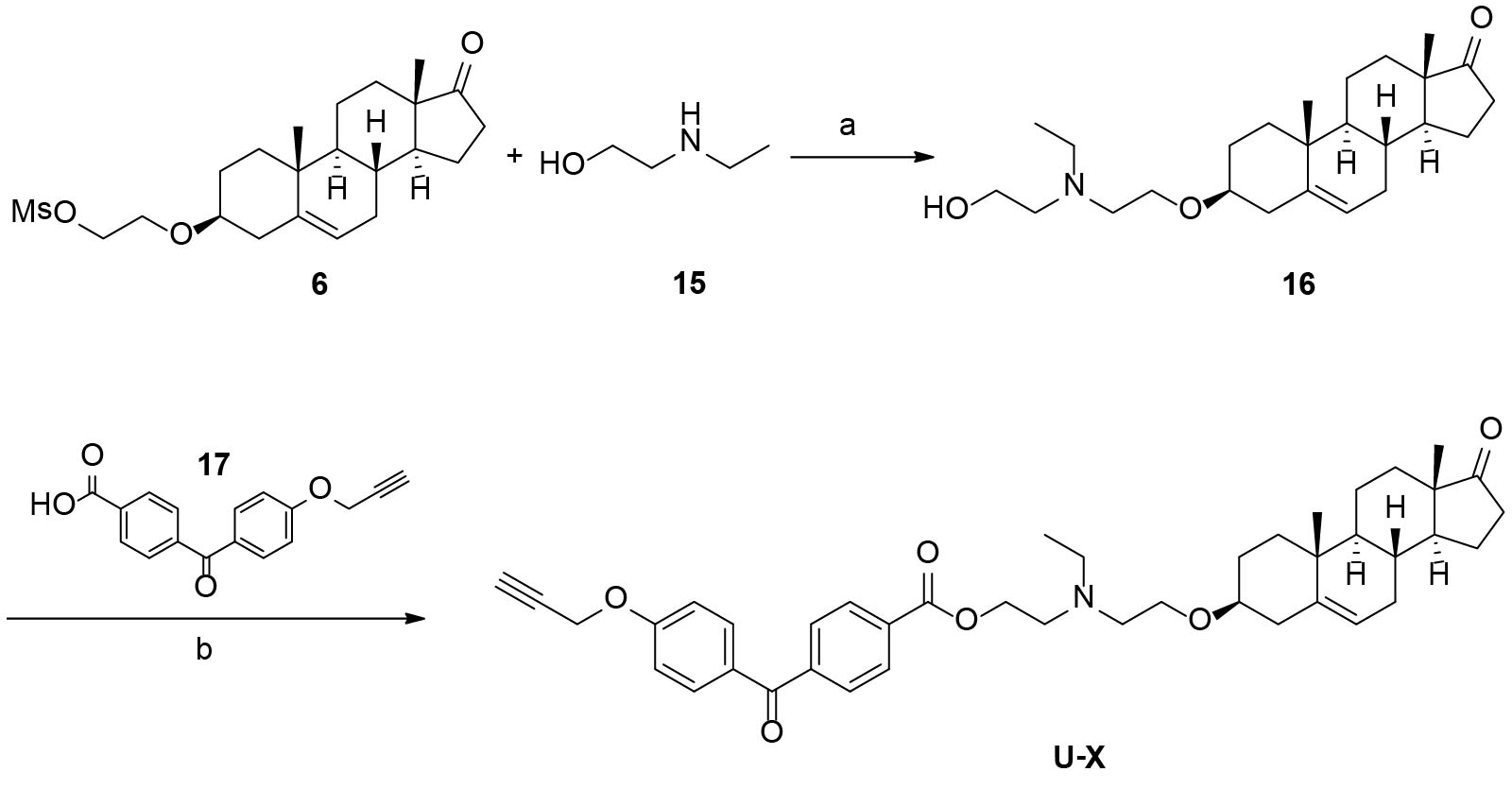


Reagents and conditions: a) toluene, 120 ^o^C; b) EDCI, DMAP, DMF, rt. Abbreviations: DMAP, 4-(dimethylamino)pyridine; EDCI, 1-ethyl-3-(3-dimethylaminopropyl)carbodiimide.

**Compound 16:** To a solution of compound **6** (200 mg, 0.48 mmol) in toluene (10 ml) was added 2-(ethylamino)ethanol (**15**, 220 mg, 2.5 mmol). After stirring for 48 hr at 120^o^C, the mixture was cooled to room temperature, and diluted with EtOAc (20 ml). The organic solution was washed with brine (2 × 4 ml), dried (Na_2_SO_4_), and concentrated. The residue was purified by flash chromatography (silica gel; Hexanes:EtOAc = 3:1) to provide compound **16** (158 mg, 0.39 mmol, 82%).

**^1^H NMR** (400 MHz, CDCl_3_) δ 5.36 (d, *J* = 4.4 Hz, 1H), 3.56-3.48 (m, 4H), 3.19-3.10 (m, 1H), 2.67 (t, *J* = 6.0 Hz, 2H), 2.65-2.59 (m, 4H), 2.45 (dd, *J* = 19.6, 8.8 Hz, 1H), 2.41-2.34 (m, 1H), 2.19 (app. t, *J* = 11.2 Hz, 1H), 2.14-2.01 (m, 2H), 1.95-1.80 (m, 4H), 1.70-1.58 (m, 3H), 1.56-1.40 (m, 3H), 1.31-1.21 (m, 2H), 1.11-0.92 (m, 2H), 1.02 (t, *J* = 7.2 Hz, 3H), 1.00 (s, 3H), 0.87 (s, 3H); **^13^C NMR** (125 MHz, CDCl_3_) δ221.61, 141.25, 121.10, 79.46, 66.70, 61.71, 59.28, 55.75, 53.26, 51.97, 50.47, 48.96, 47.80, 39.22, 37.33, 37.23, 36.09, 31.70, 31.64, 31.04, 28.47, 22.11, 20.57, 19.65, 13.78; **ESI MS:** [M+H]^+^ calcd. for C_25_H_42_NO_3_, 404.3, found 404.2.

**Compound U-X (U-photocrosslinker):** To a solution of compounds **16** (10 mg, 0.025 mmol) and **17** (14 mg, 0.05) in DMF (1 ml) was added EDCI (10 mg, 0.05 mmol) and DMAP (6 mg, 0.05 mmol). The solution was stirred overnight at room temperature, followed by dilution with EtOAc (15 ml). After washing with H_2_O (3 × 3 ml), the organic layer was dried (Na_2_SO_4_) and concentrated. The residue was purified by flash chromatography (silica gel; MeOH:CH_2_Cl_2_ = 1:10) to yield U-photocrosslinker compound **U-X** (6 mg, 0.01 mmol, 40%).

**^1^H NMR** (400 MHz, CDCl_3_) δ 8.12 (d, *J* = 8.4 Hz, 2H), 7.83-7.74 (m, 4H), 7.04 (d, *J* = 8.8 Hz, 2H), 5.32 (d, *J* = 4.8 Hz, 1H), 4.77 (d, *J* = 2.4 Hz, 2H), 4.46 - 4.39 (m, 2H), 3.65-3.55 (m, 2H), 3.17 - 3.08 (m, 1H), 3.03-2.95 (m, 2H), 2.85-2.75 (m, 2H), 2.75-2.65 (m, 2H), 2.56 (t, *J* = 2.4 Hz, 1H), 2.44 (dd, *J* = 17.2, 8.8 Hz, 1H), 2.38-2.32 (m, 1H), 2.16 (app. t, *J* = 12.8 Hz, 1H), 2.12-1.99 (m, 2H), 1.98-1.75 (m, 4H), 1.72-1.56 (m, 3H), 1.55-1.35 (m, 3H), 1.32-1.18 (m, 2H), 1.17-1.02 (m, 3H), 0.98 (s, 3H), 1.02-0.90 (m, 2H), 0.86 (s, 3H); **^13^C NMR** (100 MHz, CDCl_3_) δ 221.19, 164.82, 161.37, 151.42, 132.50, 130.33, 129.65, 129.55, 129.49, 129.16, 120.88, 114.60, 79.26, 77.63, 77.22, 76.30, 63.93, 60.45, 55.90, 54.00, 52.27, 51.72, 50.22, 49.19, 47.53, 39.03, 37.09, 36.98, 35.85, 31.46, 31.41, 30.78, 29.70, 28.27, 21.87, 21.73, 20.33, 19.39, 13.54; **ESI MS:** [M+H]^+^ calcd. for C_42_H_52_NO_6_, 666.3, found 666.2; **[α]_D_: +**4.28 (c = 0.4, CHCl_3_); calculated ClogP and pKa: 8.13 and 7.62.

D407 Supple. Info.doc
